# Supplementary material for: Balancing central control and sensory feedback produces adaptable and robust locomotor patterns in a spiking, neuromechanical model of the salamander spinal cord
Source: PLoS Comput Biol. 2025 Jan 21;21(1):e1012101. doi: 10.1371/journal.pcbi.1012101 (PMC11771899; doi:10.1371/journal.pcbi.1012101)
Supplement: S2 File — (PDF) [file pcbi.1012101.s002.pdf]

Balancing central control and sensory feedback produces adaptable and robust locomotor patterns in a spiking, neuromechanical model of the salamander spinal cord

Neuronal and synaptic parameters

Neuronal populations

The network comprises 5 different classes of neurons, namely excitatory neurons (EN), inhibitory neurons (IN), motoneurons (MN), reticulospinal neurons (RS) and propriosensory neurons (PS). The neuronal classes differ for their neuronal and synaptic properties, which were obtained based on experimental data and previous simulation studies (see Table A and Table B). Unknown properties were treated as open parameters to manually tune the activation of the network according to the known salamander locomotor patterns.

Table A. Neuronal Parameters

Neuron Type	t^*_{refr} (ms)	τ^*_{memb} (ms)	R^*_{memb} (GΩ)	$\Delta\omega$ (pA)	τ_ω (pA)
EN_{axial} [1–3]	5.0	26.8	1.6	3.0	200.0
EN_{limbs} [1–3]	5.0	37.5	2.1	0.7	500.0
IN_{axial} [1–3]	5.0	26.8	1.6	3.0	200.0
IN_{limbs} [1–3]	5.0	37.5	2.1	0.7	500.0
RS [4]	5.0	26.8	8.0	0.0	-
MN [5]	5.0	26.8	16.0	0.0	-
PS [6, 7]	50.0	26.8	0.6	0.0	-

In Table A, the value of the variables denoted by an asterisk was sampled randomly from a Gaussian distribution for each neuron of the network. The table reports the mean of the distributions. The standard deviation was set to 20% of mean value. The value of E_{rest}, E_{reset}, E_{thres} (see Eq 1 in the main text) was shared among all the neuron populations and equal to $-70.0mV$, $-70.0mV$ and $-38.0mV$ respectively. The RS, MN and PS populations were modeled with non-adaptive neurons $\Delta\omega = 0$. The PS population was modeled with a large t_{refr} value to limit its maximum firing rate according to experimental findings [6, 7]. The same effect could be obtained using highly adaptable neurons and a more physiologically-sound refractory period.

In Table A, the parameters for the synaptic variables of the model are reported (see Eq 3 and 4 in the main text). Neurons with an excitatory action acted on the AMPA and NMDA synaptic conductances. Conversely, inhibitory neurons influenced the GLYC component.

Table B. Synaptic Parameters

Synaptic Type	$E_{\text{syn}}(mV)$	$\tau_{\text{syn}}(ms)$	Neuron populations
AMPA [1, 2]	0.0	20.0	EN, RS, PS
NMDA [1, 2]	0.0	100.0	EN, RS, PS
GLYC [1, 2]	-85.0	20.0	IN, PS

Finally, the synaptic weights Δg_{syn} (see Eq 4 in the main text) were selected to replicate the patterns of activations observed in salamanders and the values are reported in Table C.

Table C. Synaptic Weights

Source	Target	Δg_{ampa}	Δg_{nmda}	Δg_{glyc}
EN_{axial}	CPG_{axial}	0.025	0.007	-
IN_{axial}	CPG_{axial}	-	-	0.036
EN_{limbs}	CPG_{limbs}	0.083	0.025	-
IN_{limbs}	CPG_{limbs}	-	-	0.128
EN_{limbs}	CPG_{axial}	0.044	0.013	-
IN_{limbs}	CPG_{axial}	-	-	0.086
RS_{axial}	CPG_{axial}	0.053	0.015	-
RS_{limbs}	CPG_{limbs}	0.083	0.025	-
PS^*_{EX}	CPG	0.025 ω_{PS}	0.007 ω_{PS}	-
PS^*_{EX}	MN	0.025 ω_{PS}	0.007 ω_{PS}	-
PS^*_{IN}	CPG	-	-	0.036 ω_{PS}
PS^*_{IN}	MN	-	-	0.036 ω_{PS}
EN	MN	0.070	0.020	-
MN	MC	0.350	0.150	-

Despite having identical neuronal properties, the IN and EN populations differ in their synaptic actions (see Table B). Together, the two populations represent the core rhythmogenic component of the network (i.e., the CPG network). For this reason, the EN and IN populations were assigned different values for their axial and limbs sub-networks [1,8]. The primary distinguishing factor between the two sub-populations lies in their adaptation time-constants and weights (see Table A and Table C). The higher adaptation time of the limb populations results in a lower frequency of the generated oscillations, in accordance with experimental findings.

Note that the synaptic weight of excitatory (PS_{EX}) and inhibitory (PS_{IN}) proprioceptive sensory neurons is scaled by the sensory feedback weight ω_{PS}. When $\omega_{PS} = 1$, the sensory neurons have the same synaptic weight as the intra-CPG connections between EX and IN neurons.

Muscle cells model

The muscle cells act as low-pass filters of the input activity (see Eq 6 in the main text). Their input is normalized in the range [0, 1] and can be interpreted as the degree of activation of the corresponding network hemisegment. The parameters for the muscle cells are reported in Table D.

Table D. Muscle cells Parameters

Parameters	Value
$\tau_{\mathrm{mc}}(ms)$	100.0
$E_{\mathrm{AMPA}_{mc}}(\#)$	1.0
$E_{\mathrm{NMDA}_{mc}}(\#)$	1.0
$E_{\mathrm{GLYC}_{mc}}(\#)$	0.0
$\tau_{\mathrm{AMPA}_{mc}}(ms)$	2.0
$\tau_{\mathrm{NMDA}_{mc}}(ms)$	10.0
$\tau_{\mathrm{GLYC}_{mc}}(ms)$	2.0

Connectivity parameters

Table E. RS connectivity

RS population	Amp	UP_0 [m]	UP_1 [m]	DW_0 [m]	DW_1 [m]
Rost	0.02	-	0.000	0.125	0.225
Midt	0.02	0.025	0.125	0.250	0.350
Endt	0.02	0.150	0.250	0.375	0.400
Pelv	0.02	0.350	0.375	0.583	0.683
Tail	0.02	0.483	0.583	0.792	0.892
Caud	0.02	0.692	0.792	1.000	-

The RS connection probabilities are given by overlapping trapezoidal distributions. The probability of a connection between neurons increases linearly from 0 to the maximum amplitude (Amp) for neurons placed in a position between UP_0 and UP_1. The probability of connections is constant and equal to Amp for connections between UP_1 and DW_0. Finally, the connection probability decreases linearly from Amp to 0 for neurons in positions between DW_0 and DW_1.

Table F. Axial connectivity

Source	Target	Side	A	σ_{up} (mm)	σ_{dw} (mm)
EN	EN	IPSI	0.5	2.00	3.00
EN	IN	IPSI	0.5	2.00	2.50
IN	EN, IN	CONTRA	0.5	2.00	3.00
EN	MN	IPSI	0.5	2.50	5.00
IN	MN	CONTRA	0.5	1.25	2.50
MN	MC	IPSI	1.0	2.50	2.50
PS	EN, IN, MN	IPSI, CONTRA	1.0	5.00	5.00

References

1. Bicanski A, Ryczko D, Cabelguen JM, Ijspeert AJ. From lamprey to salamander: an exploratory modeling study on the architecture of the spinal locomotor networks in the salamander. Biological Cybernetics. 2013;107(5):565–587. doi:10.1007/s00422-012-0538-y.

2. Bicanski A, Ryczko D, Knuesel J, Harischandra N, Charrier V, Ekeberg Ö, et al. Decoding the mechanisms of gait generation in salamanders by combining neurobiology, modeling and robotics. Biological Cybernetics. 2013;107(5):545–564. doi:10.1007/s00422-012-0543-1.

3. Dougherty KJ, Kiehn O. Firing and cellular properties of V2a interneurons in the rodent spinal cord. Journal of Neuroscience. 2010;30(1):24–37.

4. Flaive A, Ryczko D. Patch-clamp recordings in slices of telencephalon, diencephalon and rhombencephalon of salamanders. bioRxiv. 2020; p. 2020–06.

5. Chevallier S, Nagy F, Cabelguen JM. Cholinergic control of excitability of spinal motoneurones in the salamander: Cholinergic control of motoneurone excitability in salamander. The Journal of Physiology. 2006;570(3):525–540. doi:10.1113/jphysiol.2005.098970.

6. Picton LD, Bertuzzi M, Pallucchi I, Fontanel P, Dahlberg E, Björnfors ER, et al. A spinal organ of proprioception for integrated motor action feedback. Neuron. 2021;109(7):1188–1201.e7. doi:10.1016/j.neuron.2021.01.018.

7. Buchanan JT. Electrophysiological properties of identified classes of lamprey spinal neurons. Journal of Neurophysiology. 1993;70(6):2313–2325.

8. Ijspeert AJ, Crespi A, Ryczko D, Cabelguen JM. From swimming to walking with a salamander robot driven by a spinal cord model. Science. 2007;315(5817):1416–1420. doi:10.1126/science.1138353.
