# Supplementary material for: Balancing central control and sensory feedback produces adaptable and robust locomotor patterns in a spiking, neuromechanical model of the salamander spinal cord
Source: PLoS Comput Biol. 2025 Jan 21;21(1):e1012101. doi: 10.1371/journal.pcbi.1012101 (PMC11771899; doi:10.1371/journal.pcbi.1012101)
Supplement: Supplementary file 6 [file pcbi.1012101.s012.doc]

Here is the English translation of the provided Japanese text, as requested:

---

This study proposes a neuro-mechanical model of the salamander that incorporates a Central Pattern Generator (CPG) and a proprioceptive feedback (PSFB) loop related to axial bending. It investigates the characteristics of motor patterns under various parameter conditions, such as the intensity of reticulospinal (RS) drive, noise levels, the strength of PSFB, and the topology of excitation and inhibition.

I felt that the paper is highly organized with numerous analyses, providing substantial information and insights for researchers interested in the neural mechanisms of locomotion. Given the density of information, it might be beneficial to split the content into two separate papers, focusing on Open-loop and Closed-loop aspects. Particularly, the finding that excessive sensory feedback can be undesirable is likely to have a significant impact on researchers in robotics, neuroscience, and human movement rehabilitation. Alternatively, all Open-loop content could be placed in the supplementary materials. However, the decision lies with the authors, and I would not object to accepting the paper without major changes. That said, I have a few questions which I will summarize below. I hope that addressing these questions and comments will lead to minor revisions, making the paper clearer for readers. I am also looking forward to seeing the results expanded to modalities such as proprioceptive (flexion of the shoulders and waist) and exteroceptive (reaction force) being published.

**Major suggestions for improving the manuscript:**

**Q1:** The reviewer has searched for the authors' previous work online but is unclear about what is novel in this paper. Is it the examination of the effects of the five factors—EN, IN, MN, PS_ex, and PS_in? Reference [83] seems to include four factors: EN, IN, MN, and proprioceptive. Is the novelty in extending this to swimming? Or is it in the systematic investigation of the balance between CPG and PSFB using the weight ωps?

**C1:** The contributions mentioned in the title do not clearly convey the novelty or discoveries of the paper. The feature of intermediate-level abstraction is not mentioned. The fact that the neuro-model and physical model are closed-loop is also not clearly communicated. It might be better to include the intention of investigating parameters and finding an appropriate balance, for example, "intricate balance between central control and sensory feedback in producing adaptable and robust locomotor patterns in a spiking, neuro-mechanical model of the salamander locomotor simulations."

**C2:** It is unclear from the last paragraph of the Introduction whether the five questions have been addressed. Particularly for the first question, there does not seem to be a conclusion. While it may be limited for walking, it could be said that both swimming and walking can be generated.

**C3:** The feedback topology targeted in this study should also be shown in the Introduction. Until reading the Closed-loop section, it is not clear what is meant by the third question's intent. A brief explanation such as "topology concerning excitation or inhibition and ascending or descending in the motor circuit" might be sufficient.

**C4:** It is good to separate the five questions, but it takes time to understand as they do not correspond one-to-one with the structure (Open-swim, Open-walk, Close-swim). Could this be improved?

**Minor questions and comments:**

**Q2:** Please explain why the amplitude of RS firing is in pA and the noise is in mV. Isn't the noise added to the stimulation amplitude to RS neurons? The use of current notation in (5) is confusing.

**Q3:** While it was found that the impact of noise amplitude in mV is small, why wasn't the impact of noise frequency investigated?

**Q4:** Are the weights ωps for ampa, nmda, and glyc the same?

**C5:** The interaction of the factors was not investigated in this study, so it might be worth exploring in the future.

**Q5:** What determines the laterality in the "winner takes all" mechanism?

**C6:** The significance of including walking reproduction is unclear, giving an impression of incompleteness. It might be better to separate them.

**Q6:** It is stated that the influence of the inhibitory system is strong, but shouldn't the parameters of the two excitatory components be summed and compared?

**C7:** It is stated that the influence of the inhibitory system is strong, but the waveform of changes in response to RS strength in Figure 4A does not seem to match the shape in Figure 6A.

**Q7:** In Figure 7, the vibrations of the torso and tail are opposite; is this because the networks on the left side are shown? Would synchronization occur if the torso were on the right and the tail on the left? Is there a necessity to show the left networks?

**Q8:** What does "the local" (and flexible) connections between the limbs and the body refer to in lines 365? Does it mean there are both common and uncommon elements such as periodicity and a 180-degree phase lag, referred to as "local"?

**C8:** It might be helpful to mention in the caption of Figure 8 that θ_RH=10%, as in Figures S1 in S5. 

**C9:** It was also unclear why only 10% and 50% were compared and whether the intermediate values were not examined. There are many results showing triphasic patterns, so it concerns whether a different trend might exist between θ_RH=20-40%.If only 10% and 50% are shown due to space limitations, it should be noted.

**Q9:** Why is the PS range a continuous value from [0.0, 2.5] cm while the segments are discrete [0,10]? Is there a gradation in the activation within a single segment?

**C10:** The caption for Figure 9 states "The system evolves to minimize," but it seems unclear where the element of evolution is included in the model. Is it the phase oscillator?

**Q10:** Is Figure 9B an illustrative diagram of changes rather than actual simulation data? If so, it should be clearly noted.

**Q11:** The distinction between "local" and "global" in lines 567-571 is unclear. Does "local" refer to the breadth of the PS range, or does it refer to local/global networks? It seems to suggest that PS affects circuits at higher levels and rewrites the feedforward, is that correct?

**Q12:** The phrase "This is due to the incapability of the network to reach the higher rheobase angle" in lines 595-596 seems to require clarification on whether this is a limitation of the model or due to a lack of experimental data underpinning the model. An explanation for why the analysis was capped at 50% would be appreciated.

**More Minor questions and comments:**
- In Figure 2E, the threshold for θ_TH seems wide; it would be helpful to clarify the value of θ_TH. If θ_RH=θ_TH, it might be useful to state that θ_RH=θ_TH changes from 10% to 50% in the caption.
- The term "20 instances of the network, with different random seeds" seems to refer to the axial network in Figure 1A. What does "seed" refer to?
- Why are there "30 differenet instances" in Figure 5C?
- In line 363, does "In this context" refer to previous studies or the present study?
- In line 603, "was observed" appears twice.
- Figures 2E and 9A3 seem to be the same; it might be better to include Figure 9A in Figure 2. Similarly, Figure 8A might be better included in Figure 1 for a clearer explanation of the topology. It might also be easier to understand if Figures 8A and 9A were combined into a new Figure 3. In any case, figures related to the Method should precede the Results section, and those related to the results should follow the Results section for better clarity.
- What do the blue vertical lines in Figures 9 B2-B5 represent?
- The captions for Figures 11D and E are reversed.
- In the main text, abbreviations sometimes appear before they are defined; it would be good to review this.
- The term "dg_syn" in equation (4) should be consistent with "Δg_syn" in equation (9).
- Is a 1ms communication delay between Brain2 and MuJoCo FARMS appropriate? Also, does the 2ms synaptic transmission delay introduce unexpected delays in the model calculations?
- In Supplement 3, the caption for Figure S1C is reversed. Is the resonance frequency of 8Hz and damping factor of 1 used in previous studies? What is the basis for these choices?
- In Supplement 4, please clarify the reasoning behind the statement that the Reference and Simulation match, as mentioned in the caption of Figure S1B. The difference seems to be around 0.05BL/cycle, so a difference of about 20% relative to the rough Simulation value of 0.25BL/cycle seems significant.
- What is the difference between Figure S2 in Supplement 5 and Figure 9B? The presence or absence of dark green was noted, but the overall difference was unclear.
