# Supplementary material for: Balancing central control and sensory feedback produces adaptable and robust locomotor patterns in a spiking, neuromechanical model of the salamander spinal cord
Source: PLoS Comput Biol. 2025 Jan 21;21(1):e1012101. doi: 10.1371/journal.pcbi.1012101 (PMC11771899; doi:10.1371/journal.pcbi.1012101)
Supplement: Attachment — Submitted filename: response_to_reviewers.pdf [file pcbi.1012101.s013.pdf]

Authors’ Response to Reviews of

Sensory and central contributions to motor pattern generation in a spiking, neuro-mechanical model of the salamander spinal cord

A. Pazzaglia, A. Bicanski, A. Ferrario, J. Arreguit, D. Ryczko, A. Ijspeert
PLOS Computational Biology,

RC: ***Reviewers’ Comment***, AR: Authors’ Response, AC: Authors’ Changes,

1. Reviewer #1

1.1. GENERAL COMMENTS

We would like to sincerely thank the reviewers for some excellent points raised. We believe that answering to their concerns significantly improved our manuscript. In the following text, **RC** indicates the reviewers’ comments, AR indicates our responses, AC describes our changes to the paper.

RC: ***The study by Pazzaglia A, etc. presents a spiking neural network dependent neuromechanical model and investigates the role of proprioceptive sensory feedback. There is novelty that the interplay of proprioceptive feedback and CPG has been studied in many other species, but rarely salamanders. There a few points may be helpful to present the work.***

RC: ***(C1) The proposed model fails to reproduce fundamental biological features of CPG, such as oscillation in segments.***

AR: We agree that this is a fundamental biological feature of CPG that should have been discussed more in detail. While it was not explicitly stated in the text, the model is actually capable to display oscillations in the isolated spinal cord, hemicord, and even segments and hemisegments. To show this, we added simulations where we by-passed the descending RS input and directly stimulated the axial CPG network. This condition is closer to the fictive locomotion experiments where oscillations in segments and hemisegments are typically investigated by NMDA baths [1–3].

AC: Added section "Simulating fictive locomotion" in supplementary file S5.
Added fictive locomotion results as Fig. S1 in supplementary file S5.
Added mentions of these results in the introduction, conclusions and section "Swimming network".

The new Fig. S1 in file S5 shows the results from the four mentioned network architectures. In Fig.S1A, the full spinal cord (i.e.; 40 equivalent segments) was stimulated with a uniform external current of 20pA, corresponding to the average rheobase current of axial CPG neurons. The corresponding activity resulted in a travelling wave of left-right alternating oscillations along the spinal cord, resembling the ones observed during fictive swimming [2, 3]. Similarly, Fig.S1B shows that the full hemicord (i.e.; only one side of the circuit) can also independently produce oscillations. This property is a consequence of the recurrent ipsilateral excitation within the network, together with the adaptative nature of the CPG neurons. Again, this result is in accordance to fictive locomotion experiments in salamanders and lampreys [2–5].

In Fig.S1C, the network was reduced from 40 segments to only 2 equivalent segments (i.e.; from 4800 to 240

simulated neurons). The isolated spinal cord section was still able to produce left-right alternating oscillations. Additionally, the oscillations had a lower cycle duration compared to the full cord, consistently with [2]. Finally, in Fig.S1D the network was reduced to only 2 equivalent hemisegments. Again, the isolated spinal cord section was able to produce oscillations. Additionally, the hemisegmental oscillations had a lower rhythmicity, higher duty cycle and a higher frequency compared to the corresponding segmental activity. This is again in accordance with the experimental literature from fictive locomotion experiments in salamanders and lampreys [1,2]

Notably, oscillations could still be seen in the evolution of the filtered spike count even when a single segment or hemisegment of the network was considered. In this case, however, the spiking activity was sparse and more difficult to interpret (not shown). Interestingly, [2] also reports very low PTCC values for isolated segments and hemisegments in the salamander. Similarly, [6] reports a direct proportionality between the number of considered segments and the quality of the corresponding activity in the lamprey, with very low PTCC values reported for a isolated ventral roots.

Finally, the reviewer might have noticed that the original network (i.e.; the one activated by descending RS input) did not produce oscillations in absence of commissural inhibition with an RS stimulation level of 5.7pA (see Fig.S4B in Supp. File S5). Indeed, when the inhibition strength is set to 0, the axial CPG network becomes equivalent to two independent hemicord networks, which should theoretically generate oscillations by themselves. Notably, there is an active debate in the lamprey literature regarding the capability of the brain-activated network to produce hemicord oscillations [7–9]. Indeed, [8] showed that eliminating left-right reciprocal coupling could abolish the sensory-induced (and brain-driven) generation of locomotor activity, which was substituted by a tonic network activation. Interestingly, a possible explanation of this mismatch between sensory-induced and bath-induced activity could reside in a high RS activation caused by the sensory stimulation performed in [8]. Indeed, hemicord oscillations can be obtained in our model also in the RS-activated network when decreasing the external stimulation level to 4.75pA. We added another supplementary figure (see Fig, S5 in Supp. File S5) to highlight this result.

AC: Added figure of hemicord oscillations induced by descending RS drive as Fig. S5 in supplementary file S5. Added description of this result in the corresponding section of file S5 ("Effect of commissural inhibition").

Overall, we recognize the importance of discussing the capability of the network to produce oscillations at the level of segments and hemisegments. The newly added results (see section "Simulating fictive locomotion" and Figures S1, S5 in Supp. File S5) show that the proposed model aligns well with the available literature from fictive locomotion experiments in salamanders.

RC: ***(C2) The authors stated in the methods that the type and number of neurons are largely unknown and they are based their model on estimation, some from other kinds of animals. It is not impossible, or even difficult, to obtain such experimental data given current technologies. The results thus cannot lead to concrete conclusions of biological system. This may suit better as an electromechanical model for robotic control inspired by biology and simulation results showing features of such electromechanical model.***

AR: We disagree that the identification of cell types in the salamander (and other species) is a simple task, even with the current technologies. Typically the cell type classification can be done by collecting and analysing large samples of electrophysiological data (i.e. current, voltage clamp experiments) and anatomical data (for example, this work has been done in the Xenopus tadpole, see [10]). More recently, other groups have started to use genetic techniques to identify interneuron classes in the zebrafish and mouse (i.e. see [11] for a review). Both techniques require intensive research to be fully developed. Even more work is required for identifying synaptic interactions between neural classes (using pairwise recordings or optogenetic stimulation). Some of the authors in this paper have started to collect and work on identifying neural classes and their connections

in the salamander but the data collection and analysis are still ongoing [12].

Therefore, since a detailed picture of the numbers and ion channel compositions of the different cell types is still missing in the salamander we opted for an all-in-one approach to model all cell types. Using a detailed Hodgkin-Huxley model would require the tuning a high dimensional parameter space with no data available. Our model consists of a a generic spiking neuron with firing adaptation for all neural classes, which have the advantage of being computationally tractable, and having less parameters to tune. We exhaustively studied the parameter space of the model using parameter sweeps and sensitivity analyses to determine the network dynamics and the dependence on the model properties.

Despite its simplicity (or perhaps, because of it), the model can replicate several fundamental properties of locomotor spinal circuits shared across all known vertebrates (tadpole, lamprey, zebrafish, salamanders, etc., for a review, see [11]), namely:

- The CPG network comprises chains of recurrent ipsilateral excitatory neurons and commissural inhibitory neurons distributed along the spine.
- The synaptic parameters and receptors match those found across all species (glutamate, glycine)
- Direct stimulation of the CPG network results in the generation of oscillations in the full cord, full hemicord, isolated segments and isolated hemisegments, resembling fictive locomotion experiments.
- The CPG network can be activated and modulated by descending drive from RS neurons.
- The proprioceptive sensory neurons respond to stretch of the spinal cord and project to the locomotor circuits with ipsilateral excitation and contralateral inhibition.

Therefore, our model can reproduce the key locomotor properties that are conserved across vertebrate species. We believe that the model contains the core and conserved features of the vertebrate spinal circuits.

Additionally, the model's parameters describe real biological processes, and therefore allowed to formulate several predictions for experimental testing (e.g.; on the role of sensory feedback in the parameters of the traveling wave propagation and frequency control, see Section "The neuromechanical principles of frequency and phase-lag control by stretch feedback during swimming").

Therefore, we believe that our model is useful for both robotics control and to answer several biological question of locomotor control in neuroscience. Similar previous models have successfully been applied to study various biological questions, both in locomotion and other brain functions [13–15].

We believe that the revisions done to answer the previous concerns (see answer to C1) have clarified why our model is suitable to answer neuroscience questions.

RC: ***(C3) Is HH model or one fits all neural model enough to cover all different neuronal types in CPG? I am suspicious of this. But if yes, justification with experimental of literature evidences are needed.***

AR: See also previous response. We would like to point out that the selection of the neuronal model should generally be dictated by the question being addressed with the network. While it is true that the intermediate level of abstraction used in this work cannot replicate all the properties of real neurons (just like any neuronal model), our aim was to capture the core properties of the salamander's locomotor circuits. Despite its simplicity, the proposed network replicates several results from in-vitro, in-vivo and ex-vivo experiments on salamanders. This consideration justifies the use of the model as a tool to investigate properties of the

locomotor networks that are yet to (or very difficult to) be studied in the real biological systems. Moreover, the tractability of the selected neural model allows us to investigate large scale phenomena like gaits, couplings, RS drive, global sensory feedback properties, etc. Finally, the current work proposes several predictions that could drive the direction of future biological studies. The predictions include the effect of axial PS feedback on the frequency and intersegmental phase lag of the swimming patterns, the mismatch between in-vitro and in-vivo hemicord activity [8], the increased disturbance rejection capability of the closed loop network and the strong modulatory effect of commissural inhibitory connections.

No model will ever be able to replicate the complexity of a real biological system. Nonetheless, we can use models to test how much we understand the working principles of biological circuits and to make observations that could drive our future experiments.

RC: ***(C4) The authors argue that detailed bio-physical models are too computational consuming. Justification is required to show that certain details are not crucial in locomotion control and thus can be safely ignored to support authors' approach with the intermediate level of abstraction.***

AR: See the previous responses.

RC: ***(C5) The model takes information from other species like zebrafish heavily. However some known experimental findings in zebrafish, like different interneuron types and their physiological behavior, is not modeled here, but applied a one type for all approach here.***

AR: The zebrafish is only one of the multiple species (e.g.; zebrafish, tadpole, lamprey, eel, mouse) that were considered to pinpoint the features of the locomotor networks that are thought to be shared across animals, including salamanders [12].

In particular, there are two main characteristics of the zebrafish locomotor system that were mentioned and used for the model design (see also response C2):

- At their core, CPGs are composed of chains of recurrent ipsilaterally-projecting excitatory neurons and commissural inhibitory neurons distributed along the spine. This is a well established fact that is shared by all known locomotor CPG architectures (see [16] for a review).
- The (so far discovered) axial proprioceptive sensory organs respond to axial bending of the spinal cord and they project to CPGs with either ipsilateral excitatory projections (e.g.; lamprey [17, 18]) or with contralateral inhibitory projections (e.g.; lamprey [17, 18] and zebrafish [19]).

These aspects are thought to be evolutionarily conserved among several species, including salamanders [12]. Interestingly, the inclusion of these "core" properties of the CPG architecture was sufficient to replicate multiple known experimental results for the salamander locomotor circuits. Therefore, we did not deem necessary the inclusion in the model of additional non-salamander-specific properties.

RC: ***(C6) The model does a finer modular neural modeling with separated trunk and tail parts. However the muscle and sensory modeling parts seem to treat them the same. Do different body parts have similar phase delay in movement and sensitivity to stimulation at one spot? Current studies do not support the uniform motor and sensory patterns. It might require extensive experimental research to get every details accurately, but it is essential to implemented model based on what we know so far, even though limited.***

AR: In relation to the trunk and tail parts, according to the experimental evidence from [20, 21] there is no significant difference between the frequency and phase lag of the activity produced by the central pattern generator networks of the two regions. Indeed, in [20] it was shown that there is no significant correlation

between the bursting frequency of a given tail segment and its location along the spinal cord. Additionally, the study proved the existence of efficient short-distance coupling between the trunk and tail networks. These connections allow for the trunk and tail network to be controlled independently (e.g.; during walking) or in parallel (e.g.; during swimming) depending on the descending and sensory input to the locomotor networks.

Regarding the muscle properties of the model, they are not uniform along the body. Instead, the parameters for the Ekeberg muscle model [22] are scaled according to the inertial properties of the different body sections (see file S3, "Optimization of the muscle properties"). This optimization allows to scale the muscle parameters so that all the joints along the axial body can operate in a comparable dynamical regime. This is a novelty with respect to all previous modelling studies on salamanders, where muscle parameters were either constant along the body [23–26] or their selection was not described in detail [14, 27].

AC: We added a clarification of this in the "Electro-mechanical transduction" section of the Methods, in the main text.

Regarding the sensory component, as described in the section "Mechano-electrical transduction", we made the assumption that the sensitivity of the PS neurons scales along the body in relationship with the curvature normally experienced during swimming by the corresponding axial joints. This was done according to the hypothesis that the stiffer body joints will be more sensitive to stretch with respect to the flexible tail joints. The scaling also ensures that the activation of the sensory neurons happens in the same phase of the oscillation regardless of their amplitude. To the best of our knowledge, [28] is the only study that evaluated the response of axial proprioceptive sensory neurons along the body of an elongated swimmer (i.e.; the lamprey). Indeed, the mentioned work studied the sensory and motor responses to different types of bending in the yaw and sagittal planes. On the other hand, they did not investigate the presence of a gradient in sensitivity of the edge cells along the spine.

AC: We explained this concept more clearly by adding Fig.2D and rephrasing the section "Mechano-electrical transduction" in the Methods.

As stated by the reviewer, obtaining the missing data will require extensive experimental research and it is indeed a subject of active research. On the other hand, we have used extensive data from experimental and computational neuroscience in order to constrain and design the proposed neuromechanical model.

RC: ***(C7) It is true that zebrafish can recover from spinal cord injury pretty quickly, but their locomotion pattern changes comparing to pre-injury. The authors talk about salamander SCI, but provide no results concerning it. Without RS input, will the model functions just as in real animals? Is the pattern different from pre-injury?***

AR: Differently from the zebrafish, salamanders do not recover quickly from spinal cord injuries. Instead, they recover the capability to walk and swim approximately 6-8 weeks after a complete spinal transection [29, 30]. As discussed in the introduction, this is likely due to a lower level of excitability of the spinal circuits below the lesion compared to eels and lampreys, two species that are capable to swim immediately after a SCI [31–33]. In the proposed model, just like in real salamanders, removing the RS input would result in a silent network below the level of the transection.

We recognise the high relevance of exploring the relationship between the level of spinal excitability, sensory feedback and recovery from spinal cord injuries. Indeed, some recent simulation and robotics studies have started to investigate this topic [34, 35]. On the other hand, this was outside of the scope of the current work, so it was not analysed further.

AC: We added these considerations in the "Conclusions" as a future direction for our model.

RC: ***(C8) The article is lengthy and some parts could be simplified.***

AR: We agree that the inclusion of several analyses could create confusion in the reader and that some parts of the text could be simplified. For this reason, most of the considerations made for the open-loop swimming simulations were moved to the supplementary information. Similarly, the closed-loop sensitivity analysis, which lead to similar considerations with respect to the previous analyses, was also moved to the supplementary information.

AC: The following subsections were moved to Supp. File S5:

- "Effect of descending drive"
- "Effect of external noise"
- "Open loop sensitivity analysis"
- "Effect of commissural inhibition"
- "Closed loop sensitivity analysis"

We hope that removing these parts from the main text will improve its readability.

RC: ***(C9) In line 280, Fig 4C is mentioned but there is no such a figure.***

AC: The line was corrected. The corresponding section ("Effect of external noise") was moved to Supp. File S5 to improve readability.

RC: ***(C10) Extra right bracket in equation 10, making equation hard to understand if not familiar with muscle modeling.***

AC: Removed extra bracket in equation 10.

2. Reviewer #2

2.1. GENERAL COMMENTS

RC: ***This study proposes a neuro-mechanical model of the salamander that incorporates a Central Pattern Generator (CPG) and a proprioceptive feedback (PSFB) loop related to axial bending. It investigates the characteristics of motor patterns under various parameter conditions, such as the intensity of reticulospinal (RS) drive, noise levels, the strength of PSFB, and the topology of excitation and inhibition.***

RC: ***I felt that the paper is highly organized with numerous analyses, providing substantial information and insights for researchers interested in the neural mechanisms of locomotion. Given the density of information, it might be beneficial to split the content into two separate papers, focusing on Open-loop and Closed-loop aspects. Particularly, the finding that excessive sensory feedback can be undesirable is likely to have a significant impact on researchers in robotics, neuroscience, and human movement rehabilitation. Alternatively, all Open-loop content could be placed in the supplementary materials. However, the decision lies with the authors, and I would not object to accepting the paper without major changes. That said, I have a few questions which I will summarize below. I hope that addressing these questions and comments will lead to minor revisions, making the paper clearer for readers. I am also looking forward to seeing the results expanded to modalities such as proprioceptive (flexion of the shoulders and waist) and exteroceptive (reaction force) being published.***

AR: We thank the reviewer for the comments and suggestions. We agree that the paper, in its original status, had a high density of information that was affecting its readability. For this reason, we followed the reviewer's suggestion to move all the analyses made for the open-loop network in the supplementary materials. We hope that this will streamline and improve the structure the main text.

AC: All open-loop analyses were moved to Supp. File 5
Closed loop sensitivity analysis was also moved to Supp. File 5.

2.2. MAJOR QUESTIONS

RC: ***(Q1) The reviewer has searched for the authors' previous work online but is unclear about what is novel in this paper. Is it the examination of the effects of the five factors—EN, IN, MN, PS_{ex}, and PS_{IN}? Reference [83] seems to include four factors: EN, IN, MN, and proprioceptive. Is the novelty in extending this to swimming? Or is it in the systematic investigation of the balance between CPG and PSFB using the weight ω_{PS}?***

AR: While the model architecture is similar to the one in [14] (reference [83] in the original text), the current work proposes several aspects that are novel compared to that study, as well as all previous salamander simulation studies. The main novelties of our work can be summed up as follows:

1. It is the first spiking neural network model of the salamander locomotor circuits that shows full cord, full hemicord, hemisegmental and segmental oscillations (see newly added section "Simulating fictive locomotion" in supp. file S5). Previous models showed some, but not all, of these activation patterns [36, 37].
2. It is the first spiking neural network model of the salamander locomotor circuits that shows both swimming and walking gaits in a biologically-realistic neuromechanical model. Note that [14] only shows walking patterns.

3. It proposes a novel method to compute the muscle parameters of the mechanical model (see Supp. File S3 and response to Q18) and validates the resulting swimming kinematics with respect to experimental results (see Supp. File S4).
4. Similarly to [26], it proposes a simple yet powerful mechanism to selectively activate and finely control different components of the limbs and axial CPG networks (i.e.; the modular descending RS drive). Additionally, the granularity of the RS drive targeting the tail and trunk modules was increased compared to [26], leading to a greater level of controllability of the spinal output (see Figures 3, 4).
5. It provides in-depth analyses of the effect of axial PS feedback on the locomotor network, both in terms of its synaptic weight ant in terms of projection topology (see Figures 5, 7).
6. It proposes a mechanistic explanation for the frequency and phase lag control by stretch feedback (see Figure 6). The frequency increase can be explained as a spring-like effect by the stretch sensors in correspondence of regions with large curvature (see Fig 6A). The decrease in total wave lag derives from imbalanced PS-to-CPG interactions and the natural tendency of the network to produce rostro-caudal waves of activation (see Fig 6B).
7. It shows the beneficial effects of sensory feedback on the swimming performance with respect to efficiency, speed and robustness to external noise and internal parameters modification (see Figures 7, 8, 9). It also highlights the limitations of the network when its activity becomes dominated by sensory feedback.

AC: We added specific mentions of the novelties of this work by modifying the title, abstract, introduction and discussions (for more details, see responses to C1, C2, C4).

2.3. MAJOR COMMENTS

RC: ***(C1) The contributions mentioned in the title do not clearly convey the novelty or discoveries of the paper. The feature of intermediate-level abstraction is not mentioned. The fact that the neuro-model and physical model are closed-loop is also not clearly communicated. It might be better to include the intention of investigating parameters and finding an appropriate balance, for example, "intricate balance between central control and sensory feedback in producing adaptable and robust locomotor patterns in a spiking, neuro-mechanical model of the salamander locomotor simulations."***

AR: We thank the reviewer for the suggestions. We agree that the previous title was not helping in conveying our messages. For this reason, we propose a new one.

AC: We changed the title to "Balancing central control and sensory feedback produces adaptable and robust locomotor patterns in a spiking, neuromechanical model of the salamander spinal cord"

RC: ***(C2) It is unclear from the last paragraph of the Introduction whether the five questions have been addressed. Particularly for the first question, there does not seem to be a conclusion. While it may be limited for walking, it could be said that both swimming and walking can be generated.***

AR: We agree that the five questions were not properly addressed in the Introduction. We added explicit references to each question in the last paragraph of the Introduction, as well as in the text and in the conclusions. Regarding the walking results, we think that the obtaining of both swimming and walking with the same network is a very relevant result of the present work. Indeed, this is the first model to show this with a spiking neural network linked to a mechanical model. We underlined this aspect more clearly in the text.

AC: Explicit considerations for all the questions were added in the last paragraph of the Introduction.
Mentions of the addressed questions were added to each section of the paper.
The questions were cited again in the conclusions to underline how they were addressed in the present work.

RC: ***(C3) The feedback topology targeted in this study should also be shown in the Introduction. Until reading the Closed-loop section, it is not clear what is meant by the third question's intent. A brief explanation such as "topology concerning excitation or inhibition and ascending or descending in the motor circuit" might be sufficient.***

AR: We thank the reviewer for this observation. We agree that the targeted topology was not properly explained in the introduction. As suggested, we added specific mentions of the different target topologies (ascending and descending, excitatory and inhibitory, local and long-range).

AC: Explicit descriptions were added at the end of the introduction and in Question 3.
Added mention of the varying feedback topology in the caption of Fig. 1C.

RC: ***(C4) It is good to separate the five questions, but it takes time to understand as they do not correspond one-to-one with the structure (Open-swim, Open-walk, Close-swim). Could this be improved?***

AR: See also (C2). We addressed this concern of the reviewer by simplifying the structure of the paper and by adding specific mentions of the addressed questions in each section of the results and discussion.

AC: Simplified open-loop section, most analyses were moved to the supplementary information.
Added explicit mentions of the questions in the introduction, results and discussion, conclusions.

2.4. MINOR QUESTIONS

RC: ***(Q2) Please explain why the amplitude of RS firing is in pA and the noise is in mV. Isn't the noise added to the stimulation amplitude to RS neurons? The use of current notation in (5) is confusing.***

AR: We assume that by "amplitude of RS firing" the reviewer means the "amplitude of the stimulation to axial RS". This is expressed in pA since it represents the external current applied to the RS neurons in order to activate them and, consequently, the locomotor network. Regarding the noise amplitude, what was being described was the noise current amplitude (in pA) multiplied by the average membrane resistance of the axial CPG neurons (1.6 GOhm). We agree that this could be confusing for the reader and that expressing the noise amplitude as a current makes it easier to compare its magnitude with respect to the external stimulation level. The figures and corresponding descriptions have been updated to show the actual noise current amplitude in pA (i.e.; the original value divided by the membrane resistance).

AC: Noise values are now represented as currents in pA.
Updated noise definition in Equation 5.

RC: ***(Q3) While it was found that the impact of noise amplitude in mV is small, why wasn't the impact of noise frequency investigated?***

AR: Noise was injected in the system as an Ornstein-Uhlenbeck random process (see Eq. 5). As such, the only parameter that could be changed was the standard deviation of the associated gaussian white noise $\xi(\sigma_{noise}, t)$. It would be interesting to study the effect of an externally-imposed periodic signal on the locomotor network. Indeed, this was studied in the lamprey when mechanically bending the tail during fictive locomotion [31]. On the other hand, the focus of this analysis was on understanding the effect of external disturbances across all frequencies, hence the choice of the random process.

AC: Modifications were made to Eq. 5 and corresponding section for better clarity

RC: ***(Q4) Are the weights ω_{PS} for ampa, nmda, and glyc the same?***

AR: The synaptic weights for the PS neurons are shown in Table S3 from S2 file. As described in Equation 9 and following paragraph, ω_{PS} is a non-dimensional weight that scales the synaptic effect of the PS neurons with respect to their reference ones. For $\omega_{PS} = 1$, the sensory neurons have the same synaptic weights (i.e.; Δg_{ampa}, Δg_{nmda}, Δg_{glyc}) as the intra-CPG connections between EX and IN neurons.

AC: For better clarity, modifications were made the description of Table S3 in the S2 file.

RC: ***(Q5) What determines the laterality in the "winner takes all" mechanism?***

AR: In that configuration the network is bi-stable and can converge to either the left-winning or right-winning configuration. The "choice" depends solely on the initial conditions of the network and on slight left-right asymmetries arising from the stochastic way the connectivity matrix of the network is built.

AC: For better clarity, this was explained in the subsection "Effect of commissural inhibition" (moved to Supp. File 5).

RC: ***(Q6) It is stated that the influence of the inhibitory system is strong, but shouldn't the parameters of the two excitatory components be summed and compared?***

AR: The choice of studying in greater detail the effect of commissural inhibition derived from the observation that Δg_{glyc} was the parameter associated with the highest total-order sensitivity indices with respect to frequency, total wave lag and periodicity of the network's activity (see section "open loop sensitivity analysis" and Fig. S3 in Supp. File S5). While modulating the ipsilateral excitation strength would also surely affect the resulting activity, for space reasons we limited our analysis to the most relevant components of the network.

RC: ***(Q7) In Figure 7, the vibrations of the torso and tail are opposite; is this because the networks on the left side are shown? Would synchronization occur if the torso were on the right and the tail on the left? Is there a necessity to show the left networks?***

AR: While not a necessity, we decided to only display the activity of one side of the axial network in order to better highlight the coordination between the different spinal cord regions (like torso and tail during trotting). When commissural inhibition is sufficiently high, the right side of the network displays an almost identical activity as the left side and it is active when the left side is silent, and vice versa (see section "Effect of commissural inhibition" and Fig. S4 in Supp. File S5). In the mentioned Fig. S4B there is an example of both sides of the network being shown (AX_L and AX_R). In most cases, this visualization is redundant as the key properties of the oscillating activity can be seen from just one of the two sides.

Regarding the question, the alternation between the torso and tail is a typical feature of the trotting gait in salamanders [38, 39]. If the left side of the torso and the right side of the tail were to be shown, their activity would appear synchronous.

RC: ***(Q8) What does "the local" (and flexible) connections between the limbs and the body refer to in lines 365? Does it mean there are both common and uncommon elements such as periodicity and a 180-degree phase lag, referred to as "local"?***

AR: The locality refers to the range of connections between the limb CPGs and the axial CPGs. In principle, it is possible to force the axial activity to be synchronized with the limb activity by imposing long limb-to-body connections. That is, by connecting the forelimb CPGs to the entire trunk network and by connecting the hindlimb CPGs to the entire tail network. This hypothesis was introduced in [40] but it was later deemed

unlikely based on indirect observations from ventral root recordings [26, 41]. Therefore, there is nowadays a consensus on considering only short-range (i.e.; local) connections between the limbs and axial networks.

AC: For better clarity, modifications were made to the sections "Network model" and "Walking network" when describing the limb-to-body projections.

RC: ***(Q9) Why is the PS range a continuous value from [0.0, 2.5] cm while the segments are discrete [0,10]? Is there a gradation in the activation within a single segment?***

AR: As explained in the section "Network model" of the methods, neurons of the axial network are arranged uniformly in a continuous two-dimensional sheet without explicit segmental boundaries. Each neuron is assigned a coordinate specifying its position along the spine (from 0cm to 10cm), allowing to define position-dependent connections. Since the salamander spinal cord exhibits 40 vertebrae and ventral roots, equally spaced consecutive sections of the network can be viewed to represent a spinal segment (henceforth termed an equivalent segment), with a length of 2.5mm. The notion of equivalent segment is therefore only used for evaluating the performance of the network but it has no effect on the way the network and its connections are built.

In practice, since neurons are uniformly distributed along the spine, the range of connections (like the PS range in this case) can be a continuous value and not necessarily a multiple of the equivalent segment length.

AC: This was better clarified in the section "Network model" of the methods.

RC: ***(Q10) Is Figure 9B an illustrative diagram of changes rather than actual simulation data? If so, it should be clearly noted.***

AR: Indeed, that is an illustrative diagram to explain our mechanistic explanation of phase-lag control by stretch feedback. We changed the title of the Figure as “Illustrative diagrams showing the mechanism of frequency and phase lag control by stretch feedback.". In addition we revised the related main text and caption of this figure to better convey our message.

AC: Modifications were made to Fig.6 (previously Fig.9) and Fig.S8 in Supp. File 5.
Several modifications were also made to the section "The neuromechanical principles of frequency and phase-lag control by stretch feedback during swimming" to better convey our message.

RC: ***(Q11) The distinction between "local" and "global" in lines 567-571 is unclear. Does "local" refer to the breadth of the PS range, or does it refer to local/global networks? It seems to suggest that PS affects circuits at higher levels and rewrites the feedforward, is that correct?***

AR: As correctly pointed out, the concept of "local" and "global" were not well explained in this paragraph. Here, "local" refers to an effect that can be observed at a segmental level. Indeed, the frequency increase can be explained as the effect of an equivalent spring accelerating the network dynamics in response to a high local curvature (see Fig 6A).

Conversely, "global" refers to an effect that can only be explained when looking at the activity of the whole network. In this regard, the modulation of intersegmental phase lag by PS feedback cannot be justified when considering a single segment but it is a consequence of the tendency of PS feedback to reduce the overlap between PS neurons activity and CPG activity (see Fig 6B).

AC: Several modifications were made to the section "The neuromechanical principles of frequency and phase-lag control by stretch feedback during swimming" to better convey our message.

RC: ***(Q12) The phrase "This is due to the incapability of the network to reach the higher rheobase angle" in***

lines 595-596 seems to require clarification on whether this is a limitation of the model or due to a lack of experimental data underpinning the model. An explanation for why the analysis was capped at 50% would be appreciated.

AR: See also response to (C9). At the lower limit of the stability region (i.e.; for low drives), the network produces unpatterned oscillations (see Fig.S2B in Supp. File S5, upper plot). In this case the network firing is sparse and it does not lead to a strong muscle activation on either side of the body. Conversely, at the higher limit of the stability region (i.e.; for high drives), the network produces bilateral tonic activity (see Fig.S2B in Supp. File S5, lower plot). In this case the muscles on the two sides of each joint show a similar level of co-contraction. In both cases, the corresponding joint angles are very low and they are less likely to reach the minimum PS activation angle (i.e.; θ_{RH}) when the rheobase angle ratio is increased from 10% to 50%, thus preventing feedback from exerting its effect on the network. Note that we introduced the term "rheobase angle ratio" $\theta_{RH}^{\%}$ to better highlight the quantity being modified (more details in response to C11).

AC: We rephrased the mentioned paragraph in section "Intermediate levels of sensory feedback improve the swimming performance"

RC: ***(Q13) The term "20 instances of the network, with different random seeds" seems to refer to the axial network in Figure 1A. What does "seed" refer to?***

AR: The network connectivity is built according to probability distributions that take into account the type, side and distance of the neurons (e.g. EX neurons connecting to ipsilateral IN neurons with an exponentially decaying probability, see Eq. 8). Generating the network with two different seeds for the random number generator (i.e.; random seeds) will create two different connectivity matrices (although built according to the same rules). Each distinct connectivity matrix is therefore the result of a given random seed and it is referred to as "instance" of the network.

All the analyses in the paper are repeated for multiple instances of the network (either 20 or 30) to ensure that the results are a propriety of the model architecture rather than the result of a "lucky" connectivity definition.

AC: For better clarity, all mentions of "instances of the network" were substituted with "instances of the network, built with different seeds for the random number generator".

RC: ***(Q14) Why are there "30 differenet instances" in Figure 5C?***

AR: See answer to Q13. The sensitivity analysis was carried out for 30 instances of the model, each one built according to the same stochastic rules. The violin plots and boxplots represent the distribution of total-order sensitivity indices for the 30 resulting sensitivity analyses (see also the "Sensitivity analysis" section in the methods).

RC: ***(Q15) In line 363, does "In this context" refer to previous studies or the present study?***

AR: In the context of the described mismatch between in-vitro and in-vivo activity of the tail network.

AC: Rephrased corresponding paragraph in section "Walking network" of the results.

RC: ***(Q16) What do the blue vertical lines in Figures 9 B2-B5 represent?***

AR: Indeed, these lines (and the yellow arrows) were not properly explained in the figure caption. The lines represent the start/end of the unbalanced inhibition for ascending PS_{IN} and caudally for descending PS_{IN}. We modified the figure and the caption to (1) simplify the mechanistic explanation (i.e. removed brown areas, which were not useful) and (2) improved the text. For the specific comments, we included the following lines in the caption of the figure, which we hope now better explain the meaning of these lines: “The light blue

arrows represent the PS_{IN} projections in the ascending (B2-B3) and descending (B4-B5) directions to the closest CPG neurons on the opposite sides, respectively. These indicate the end/start of a region of unbalanced (i.e.; not uniformly distributed across the network) inhibition from PS_{IN} to the CPGs (red areas). This imbalance in PS_{IN} inhibition causes a delay shift in the activations of the corresponding CPGs according to the direction indicated by the yellow arrows."

AC: Modifications in Fig.6 (previously figure 9) and Fig. S8 of Supp. File S5.
Several modifications to the section "The neuromechanical principles of frequency and phase-lag control by stretch feedback during swimming" to better convery our message.

RC: ***(Q17) Is a 1ms communication delay between Brain2 and MuJoCo FARMS appropriate? Also, does the 2ms synaptic transmission delay introduce unexpected delays in the model calculations?***

AR: A timestep of 1ms is typically used to simulate networks of Integrate and Fire neurons [14,42] and mechanical models [14, 27, 43]. Brian2 has a built-in solver that automatically takes care of the integration of synapses with transmission delays so it is robust to their use in the model. Additionally, comparable delays have been used in previous models [14, 36]

RC: ***(Q18) In Supplement 3, the caption for Figure S1C is reversed. Is the resonance frequency of 8Hz and damping factor of 1 used in previous studies? What is the basis for these choices?***

AC: Corrected caption

AR: The parameters were not used in previous studies on salamanders as the described optimization method is a novelty of the current work. In [44], the role of mechanical resonance in a simplified single-joint mechanical model of the lamprey was explored. The authors of that work studied similar ranges for the resonance frequency (although scaled for the slower oscillations in the lamprey) and damping factors. The choice of the parameters was based on the following considerations.

For the resonance frequency, a value of 8Hz was chosen to be sufficiently higher than the maximum frequency displayed by salamanders during swimming ($\approx 4.5Hz$ [30,38]). This ensures that the muscles are sufficiently stiff to produce movements across all frequencies displayed during normal behaviors. On the other hand, with very high resonant frequencies the joints would result too stiff to be influenced by external factors such as the inertial, frictional and hydrodynamic forces acting on the body. This configuration would effectively result in a position-controlled model where the joint angles are identically equal to the resting angle of the Ekeberg muscle

$$\theta_i = -\frac{\alpha_i M_{diff_i}}{\beta_i(\gamma_i + M_{sum_i})} \tag{1}$$

Therefore, the final choice was a trade-off between controllability of the model and responsiveness to external factors (water, friction, inertial forces).

AC: Modifications to the resonance frequency selection description in file S3

For the damping factor, the value was chosen to avoid unwanted system responses. As described in the supplementary file S3, lower damping values led to uncontrolled motion in the caudal joints when the rostral ones were activated, similarly to the chaotic dynamics of a double pendulum [45]. Conversely, higher damping values reduced the amplitude of the generated oscillations and slowed down the muscle dynamics. Finally, similar values of damping factors (comprising 1.0) were studied in [44].

AC: Modifications to the damping factor selection description in file S3

As discussed in the text, the choice of the parameters was based on heuristic considerations and future studies could refine their estimation based on new experimental findings.

RC: ***(Q19) What is the difference between Figure S2 in Supplement 5 and Figure 9B? The presence or absence of dark green was noted, but the overall difference was unclear.***

AR: Figure 6B (previously Fig.9B) describes the case with inhibitory PS feedback (PS_{IN}). Figure S6 in File S5 describes the case with excitatory PS feedback (PS_{EX}). We made several modifications in the main text, the related figures and their captions, to clarify this and other misunderstandings related to this part. See also responses to Q10, Q11, Q16.

AC: Several modifications to the figures and corresponding section "The neuromechanical principles of frequency and phase-lag control by stretch feedback during swimming".

2.5. MINOR COMMENTS

RC: ***(C5) The interaction of the factors was not investigated in this study, so it might be worth exploring in the future.***

AR: The reviewer is likely referring to the interactions between ω_{PS}, Δg_{ampa}, Δg_{nmda} and Δg_{glyc} (mentioned in Q4). The interaction of these factors was arguably extensively discussed in the text. Indeed, the open-loop sensitivity analysis highlighted the sensitivity of the network to several parameters, including Δg_{ampa}, Δg_{nmda} and Δg_{glyc} (see section "Open loop sensitiviy analysis" and Fig. S3C in Supp. File 5). Having noticed that Δg_{glyc} (i.e.; the strength of commissural inhibition) is the parameter that mostly influences the open loop network, we studied its effects more in details in the successive section (see section "Effect of commissural inhibition" and Fig. S4 in Supp. File 5).

For the closed loop network, studying the effect of modulating ω_{PS} (and other PS properties) was the main focus of most analyses in the text (see Figures 5-9 and corresponding sections). The interplay between Δg_{glyc} and ω_{PS} was studied again to compare the results with the open loop network (see Fig. 9A,B). Finally, the interaction of all the factors mentioned by the reviewer (ω_{PS}, Δg_{ampa}, Δg_{nmda} and Δg_{glyc}) was investigated with the closed loop sensitivity analysis (see section "Closed loop sensitivity analysis" and Fig. S6 in Supp. File S5).

Overall, we think that the interaction of the factors was extensively investigated in this study. On the other hand, we agree that future studies might address other parameters of the system that were not modified in this work. Examples may include the modulation of the excitatory connections, the range of the CPG projections, the topology of the descending RS drive or the inclusion of supraspinal feedback loops.

RC: ***(C6) The significance of including walking reproduction is unclear, giving an impression of incompleteness. It might be better to separate them.***

AR: See also response to (Q1). We agree that the walking network was not studied in depth and we understand the reasons for the comment of the reviewer. On the other hand, the property of generating both swimming and walking patterns with a unified network architecture is a fundamental feature of the salamander spinal cord circuits [12]. Additionally, this is the first spiking neural network model of the salamander locomotor circuits that shows both swimming and walking patterns in a biologically-realistic neuro-mechanical model, making this an important element of novelty for the paper.

Therefore, we would like to keep the walking reproduction results, if possible. This model could be the

starting point for successive works that could investigate deeper into the inter and intra-limb coordination of the salamander locomotor network (see section "Limitations of the model").

RC: ***(C7) It is stated that the influence of the inhibitory system is strong, but the waveform of changes in response to RS strength in Figure 4A does not seem to match the shape in Figure 6A.***

AR: Figure S2A in Supp. File S5 (previously Fig.4A) displays the modulation of periodicity (PTCC), frequency (f_{neur}) and total wave lag (TWL) depending on the level of external stimulation to the axial RS neurons. Figure S2A in Supp. File S5 (previously Fig. 6A) displays the modulation of the same metrics as a function of the strength of commissural inhibition, while keeping a constant level of external RS stimulation (5.7pA).

Since the quantity being varied is different in the two cases (RS stimulation and commissural inhibition strength, respectively), there is no reason why their respective waveforms should match.

The statement that "the influence of the inhibitory system is strong" is justified by the high level of modulation of the considered metrics depending on the strength of the commissural inhibition (see Fig. S3C and Fig. S4 in Supp. File S5).

AC: We added a comment about the constant RS drive in Fig.S4 to better highlight this aspect.

RC: ***(C8) It might be helpful to mention in the caption of Figure 8 that θ_{RH}=10%, as in Figures S1 in S5.***

AR: As stated in the section "Mechano-electrical transduction" of the methods, unless stated otherwise. the rheobase angle ratio for each segment was set to 10% (see also response to C11). Note that we introduced the term "rheobase angle ratio" $\theta^{\%}_{RH}$ to better highlight the quantity being modified (more details in response to C11)

AC: For better clarity, we added an explicit mention of the value of $\theta^{\%}_{RH}$ in all the closed loop figures of the main text (Figures 5,6,7,8,9) and supplementary results (Figures S7, S9, S10, S11, S12, S13 in Supp. File 5).

RC: ***(C9) It was also unclear why only 10% and 50% were compared and whether the intermediate values were not examined. There are many results showing triphasic patterns, so it concerns whether a different trend might exist between θ_{RH}=20-40%.If only 10% and 50% are shown due to space limitations, it should be noted.***

AR: The value of $\theta^{\%}_{RH}$ influences the fraction of the angles evolution for which the PS neurons are active (see the newly added Fig. 2D). Note that we introduced the term "rheobase angle ratio" $\theta^{\%}_{RH}$ to better highlight the quantity being modified (more details in response to C11) All results in the main text are reported for $\theta^{\%}_{RH} = 10\%$, which is the lowest threshold angle we considered. When using higher $\theta^{\%}_{RH}$ values, we observed similar (although less marked) trends for all the analyses. This includes the effect of:

- Feedback topology (see Fig. 5 and Fig. S7 in Supp. file S5),
- Stimulation amplitude (see Fig. 7 and Fig. S9 in Supp. file S5),
- Noise level (see Fig. 8A,B and Fig. S12 in Supp. file S5) and
- Commissural inhibition strength (see Fig. 9 and Fig. S13 in Supp. file S5).

Intermediate $\theta^{\%}_{RH}$ values (e.g. 30%) were tested for all the analyses but they showed the same trends found in the already provided plots. Indeed, we did not find any additional triphasic pattern depending on the value of $\theta^{\%}_{RH}$. The inclusion of additional figures for intermediate $\theta^{\%}_{RH}$ values was therefore omitted purely for space reasons.

AC: As suggested by the reviewer, we justified the exclusions of results for intermediate $\theta_{RH}^{\%}$ values in the section "Mechano-electrical transduction" of the Methods.

RC: ***(C10) The caption for Figure 9 states "The system evolves to minimize," but it seems unclear where the element of evolution is included in the model. Is it the phase oscillator?***

AR: This is similar to a previous question (Q10). This figure is purely a sketch (not generated via simulations and not via optimization). This sentence was meant to explain that the network with added feedback reduces (minimises) the unbalanced inhibition to the CPG neurons. We removed this sentence to avoid the possible confusion with optimization algorithms and minimization.

In general, using this figure (and related text) we tried to provide a mechanistic rationale for explaining the effects of ascending/descending feedback on the network frequency and wave lag reported earlier in the text (see Fig.5).

AC: Several parts of the section "The neuromechanical principles of frequency and phase-lag control by stretch feedback during swimming" and the related Fig.6 (previously Fig.9) were modified to convey our message more clearly.

RC: ***(C11) In Figure 2E, the threshold for θ_{TH} seems wide; it would be helpful to clarify the value of θ_{TH}. If θ_{RH}=θ_{TH}, it might be useful to state that θ_{RH}=θ_{TH} changes from 10% to 50% in the caption.***

AR: We agree that the difference between θ_{RH} and θ_{TH} was not properly explained and that the use of these two terms could be confusing. For this reason, we decided to substitute the term "threshold angle" with the term "rheobase angle", which better conveys the relationship between the joint angle and the rheobase current of the PS neurons. Similarly, we found useful to distinguish between the "rheobase angle" θ_{RH} and the "rheobase angle ratio" $\theta_{RH}^{\%}$. The former, as discussed, is the angle value for which the PS neurons at a given segment become active and it changes along the axial network proportionally to the oscillation amplitudes observed in swimming salamanders for the corresponding axial segment [39] (see section "Mechano-electrical transduction"). The latter expresses the ratio between the rheobase angle and the corresponding joint's oscillation angle. Notably, the rheobase angle ratio is constant along the axial network and it is equal to 10% in most simulations.

Regarding Figure 2E, the panel shows the angle-current relationship in the analogy with proportional control discussed in the section "Mechano-electrical transduction". As such, the x-axis shows a generic angle θ without specifying its value. Therefore, the plot is independent of the chosen value of $\theta_{RH}^{\%}$. We agree that this plot in isolation might be confusing for the reader. For this reason the panel D in the figure was substituted with a schematic explanation of $\theta_{RH}^{\%}$ and the PS neurons response during the evolution of a joint's angle.

AC: Modified figure 2D to highlight the relationship between the joint angle and the rheobase angle θ_{RH}.
Modified section "Mechano-electrical transduction" to provide the definition of θ_{RH} and $\theta_{RH}^{\%}$.
Substituted term "threshold angle" with "rheobase angle" for consistency across the figures and discussions.

RC: ***(C12) In line 603, "was observed" appears twice.***

AC: Removed repetition

RC: ***(C13) Figures 2E and 9A3 seem to be the same; it might be better to include Figure 9A in Figure 2. Similarly, Figure 8A might be better included in Figure 1 for a clearer explanation of the topology. It might also be easier to understand if Figures 8A and 9A were combined into a new Figure 3. In any case, figures related to the Method should precede the Results section, and those related to the results should follow the Results section for better clarity.***

AR: We thank the reviewer for the valid suggestions. We made several changes to the figures in order to improve the readability and clarity of the paper.

AC: In Fig. 1C (and caption), we added an explicit reference to the analysis of the PS topology.
We removed the panel from Fig. 9A3 (now Fig. 6) and just kept the one in Figure 2E.
We substituted Fig. 2D with a schematic of the PS feedback activity depending on the joint angle.
We removed the panel from Fig. 8A (now Fig. 5) to only present the results of the corresponding section.

RC: ***(C14) The captions for Figures 11D and E are reversed.***

AC: Modified Fig. 11 (now Fig.8) and corresponding caption.

RC: ***(C15) In the main text, abbreviations sometimes appear before they are defined; it would be good to review this.***

AC: We made sure to introduce abbreviations before using them.

RC: ***(C16) The term "dg_{syn}" in equation (4) should be consistent with "$\Delta g_s yn$" in equation (9).***

AR: As described in the corresponding paragraph, the term $\frac{dg_{syn}}{dt}$ in Eq. 4 represents the temporal derivative of the synaptic conductance g_{syn}. Conversely, the term Δg_{syn} represents the impulsive increase of g_{syn} when and input spike from a synapse of type "syn" is received (i.e.; the synaptic weight). Similarly, the term in Eq. 9 represents the synaptic weight of PS neurons and it is therefore indicated as Δg_{syn}, consistently with the previous notation.

RC: ***(C17) In Supplement 4, please clarify the reasoning behind the statement that the Reference and Simulation match, as mentioned in the caption of Figure S1B. The difference seems to be around 0.05BL/cycle, so a difference of about 20% relative to the rough Simulation value of 0.25BL/cycle seems significant.***

AR: As underlined by the reviewer, it is not correct to say that the simulation matches the experimental data exactly. On the other hand, real animals display a large degree of variability in their kinematics. The variability involves the evolution of joint angles and body lateral displacements (see the blue band in Fig. S1B), as well as the frequency and speed of locomotion [39, 46, 47]. Indeed, the variability observed in salamanders for the relationship between frequency and speed is arguably much larger than the 20% difference pointed out by the reviewer (e.g.; see Fig. 6 in [46]).

Overall, the main message of the discussion was that, despite the simplicity of the hydrodynamic model, there is a surprising similarity between the simulated and reference kinematics, both with respect to the lateral displacements (Fig. S1A) and with respect to the swimming speed (Fig. S1B).

AC: Modified caption and text description of Fig S1 in file S4.

References

[1] Cangiano L, Grillner S. Fast and Slow Locomotor Burst Generation in the Hemispinal Cord of the Lamprey. Journal of Neurophysiology. 2003;89(6):2931–2942. doi:10.1152/jn.01100.2002.

[2] Ryczko D, Charrier V, Ijspeert A, Cabelguen JM. Segmental Oscillators in Axial Motor Circuits of the Salamander: Distribution and Bursting Mechanisms. Journal of Neurophysiology. 2010;104(5):2677–2692. doi:10.1152/jn.00479.2010.

[3] Ryczko D, Knüsel J, Crespi A, Lamarque S, Mathou A, Ijspeert AJ, et al. Flexibility of the axial central pattern generator network for locomotion in the salamander. Journal of Neurophysiology. 2015;113(6):1921–1940. doi:10.1152/jn.00894.2014.

[4] Cangiano L. Mechanisms of Rhythm Generation in a Spinal Locomotor Network Deprived of Crossed Connections: The Lamprey Hemicord. Journal of Neuroscience. 2005;25(4):923–935. doi:10.1523/JNEUROSCI.2301-04.2005.

[5] Cangiano L, Hill R, Grillner S. The hemisegmental locomotor network revisited. Neuroscience. 2012;210:33–37.

[6] Buchanan JT. The roles of spinal interneurons and motoneurons in the lamprey locomotor network. Progress in brain research. 1999;123:311–321.

[7] Jackson AW, Horinek DF, Boyd MR, McClellan AD. Disruption of Left–Right Reciprocal Coupling in the Spinal Cord of Larval Lamprey Abolishes Brain-Initiated Locomotor Activity. Journal of Neurophysiology. 2005;94(3):2031–2044. doi:10.1152/jn.00039.2005.

[8] Messina J, St Paul A, Hargis S, Thompson WE, McClellan AD. Elimination of left-right reciprocal coupling in the adult lamprey spinal cord abolishes the generation of locomotor activity. Frontiers in Neural Circuits. 2017;11:89.

[9] Cangiano L, Grillner S. Commentary: elimination of left-right reciprocal coupling in the adult lamprey spinal cord abolishes the generation of locomotor activity. Frontiers in Neural Circuits. 2018;12:34.

[10] Roberts A, Li WC, Soffe SR. How neurons generate behaviour in a hatchling amphibian tadpole: an outline. Frontiers in behavioral neuroscience. 2010;4:1535.

[11] Wilson AC, Sweeney LB. Spinal cords: Symphonies of interneurons across species. Frontiers in Neural Circuits. 2023;17:1146449.

[12] Ryczko D, Simon A, Ijspeert AJ. Walking with Salamanders: From Molecules to Biorobotics. Trends in Neurosciences. 2020;43(11):916–930. doi:10.1016/j.tins.2020.08.006.

[13] Kozlov A, Huss M, Lansner A, Kotaleski JH, Grillner S. Simple cellular and network control principles govern complex patterns of motor behavior. Proceedings of the National Academy of Sciences. 2009;106(47):20027–20032. doi:10.1073/pnas.0906722106.

[14] Harischandra N, Knuesel J, Kozlov A, Bicanski A, Cabelguen JM, Ijspeert AJ, et al. Sensory Feedback Plays a Significant Role in Generating Walking Gait and in Gait Transition in Salamanders: A Simulation Study. Frontiers in Neurorobotics. 2011;5. doi:10.3389/fnbot.2011.00003.

[15] Kozlov AK, Kardamakis AA, Hellgren Kotaleski J, Grillner S. Gating of steering signals through phasic modulation of reticulospinal neurons during locomotion. Proceedings of the National Academy of Sciences. 2014;111(9):3591–3596.

[16] Grillner S, El Manira A. Current Principles of Motor Control, with Special Reference to Vertebrate Locomotion. Physiological Reviews. 2020;100(1):271–320. doi:10.1152/physrev.00015.2019.

[17] Grillner S, Williams T, Lagerbäck PÅ. The edge cell, a possible intraspinal mechanoreceptor. Science. 1984;223(4635):500–503.

[18] Viana Di Prisco G, Walle´n P, Grillner S. Synaptic effects of intraspinal stretch receptor neurons mediating movement-related feedback during locomotion. Brain Research. 1990;530(1):161–166. doi:10.1016/0006-8993(90)90675-2.

[19] Picton LD, Bertuzzi M, Pallucchi I, Fontanel P, Dahlberg E, Björnfors ER, et al. A spinal organ of proprioception for integrated motor action feedback. Neuron. 2021;109(7):1188–1201.e7. doi:10.1016/j.neuron.2021.01.018.

[20] Charrier V, Cabelguen JM. Fictive rhythmic motor patterns produced by the tail spinal cord in salamanders. Neuroscience. 2013;255:191–202. doi:10.1016/j.neuroscience.2013.10.020.

[21] Cabelguen JM, Charrier V, Mathou A. Modular functional organisation of the axial locomotor system in salamanders. Zoology. 2014;117(1):57–63.

[22] Ekeberg Ö. A combined neuronal and mechanical model of fish swimming. Biological cybernetics. 1993;69(5-6):363–374.

[23] Liu Q, Zhang Y, Wang J, Yang H, Hong L. Modeling of the neural mechanism underlying the terrestrial turning of the salamander. Biological Cybernetics. 2020;114(3):317–336. doi:10.1007/s00422-020-00821-1.

[24] Liu Q, Wang JZ. Modeling and analysis of a new locomotion control neural networks. Biological Cybernetics. 2018;112(4):345–356. doi:10.1007/s00422-018-0758-x.

[25] Liu Q, Yang H, Zhang J, Wang J. A new model of the spinal locomotor networks of a salamander and its properties. Biological Cybernetics. 2018;112(4):369–385. doi:10.1007/s00422-018-0759-9.

[26] Knüsel J, Crespi A, Cabelguen JM, Ijspeert AJ, Ryczko D. Reproducing Five Motor Behaviors in a Salamander Robot With Virtual Muscles and a Distributed CPG Controller Regulated by Drive Signals and Proprioceptive Feedback. Frontiers in Neurorobotics. 2020;14. doi:10.3389/fnbot.2020.604426.

[27] Harischandra N, Cabelguen JM, Ekeberg Ö. A 3D Musculo-Mechanical Model of the Salamander for the Study of Different Gaits and Modes of Locomotion. Frontiers in Neurorobotics. 2010;4. doi:10.3389/fnbot.2010.00112.

[28] Hsu LJ, Zelenin PV, Grillner S, Orlovsky GN, Deliagina TG. Intraspinal stretch receptor neurons mediate different motor responses along the body in lamprey. Journal of Comparative Neurology. 2013;521(16):3847–3862.

[29] Doyle L, Stafford P, Roberts B. Recovery of locomotion correlated with axonal regeneration after a complete spinal transection in the eel. Neuroscience. 2001;107(1):169–179.

[30] Chevallier S, Landry M, Nagy F, Cabelguen JM. Recovery of bimodal locomotion in the spinal-transected salamander, Pleurodeles waltlii. Eur J Neurosci. 2004;20:1995–2007.

[31] Wallén P. Spinal mechanisms controlling locomotion in dogfish and lamprey. Acta physiologica Scandinavica Supplementum. 1982;503:1–45.

[32] Wallén P, Williams T. Fictive locomotion in the lamprey spinal cord in vitro compared with swimming in the intact and spinal animal. The Journal of physiology. 1984;347(1):225–239.

[33] Gray J. Studies in animal locomotion: IV. The neuromuscular mechanism of swimming in the eel. Journal of Experimental Biology. 1936;13(2):170–180.

[34] Thandiackal R, Melo K, Paez L, Herault J, Kano T, Akiyama K, et al. Emergence of robust self-organized undulatory swimming based on local hydrodynamic force sensing. Science robotics. 2021;6(57):eabf6354.

[35] Hamlet C, Fauci L, Morgan JR, Tytell ED. Proprioceptive feedback amplification restores effective locomotion in a neuromechanical model of lampreys with spinal injuries. Proceedings of the National Academy of Sciences. 2023;120(11):e2213302120. doi:10.1073/pnas.2213302120.

[36] Bicanski A, Ryczko D, Cabelguen JM, Ijspeert AJ. From lamprey to salamander: an exploratory modeling study on the architecture of the spinal locomotor networks in the salamander. Biological Cybernetics. 2013;107(5):565–587. doi:10.1007/s00422-012-0538-y.

[37] Knüsel J. Modeling a diversity of salamander motor behaviors with coupled abstract oscillators and a robot [PhD Thesis]. EPFL; 2013. Available from: `https://infoscience.epfl.ch/record/185992`.

[38] Delvolvé I, Bem T, Cabelguen JM. Epaxial and Limb Muscle Activity During Swimming and Terrestrial Stepping in the Adult Newt, *Pleurodeles waltl*. Journal of Neurophysiology. 1997;78(2):638–650. doi:10.1152/jn.1997.78.2.638.

[39] Karakasiliotis K, Thandiackal R, Melo K, Horvat T, Mahabadi NK, Tsitkov S, et al. From cineradiography to biorobots: An approach for designing robots to emulate and study animal locomotion. Journal of the Royal Society Interface. 2016;13(119). doi:10.1098/rsif.2015.1089.

[40] Ijspeert AJ, Crespi A, Ryczko D, Cabelguen JM. From swimming to walking with a salamander robot driven by a spinal cord model. Science. 2007;315(5817):1416–1420. doi:10.1126/science.1138353.

[41] Cabelguen JM, Ijspeert A, Lamarque S, Ryczko D. Axial dynamics during locomotion in vertebrates. In: Progress in Brain Research. vol. 187. Elsevier; 2010. p. 149–162. Available from: `https://linkinghub.elsevier.com/retrieve/pii/B9780444536136000101`.

[42] Knüsel J, Bicanski A, Ryczko D, Cabelguen JM, Ijspeert AJ. A salamander's flexible spinal network for locomotion, modeled at two levels of abstraction. Integrative and Comparative Biology. 2013;53(2):269–282. doi:10.1093/icb/ict067.

[43] Nyakatura JA, Melo K, Horvat T, Karakasiliotis K, Allen VR, Andikfar A, et al. Reverse-engineering the locomotion of a stem amniote. Nature. 2019;565(7739):351. doi:10.1038/s41586-018-0851-2.

[44] Tytell ED, Hsu CY, Fauci LJ. The role of mechanical resonance in the neural control of swimming in fishes. Zoology. 2014;117(1):48–56. doi:10.1016/j.zool.2013.10.011.

[45] Shinbrot T, Grebogi C, Wisdom J, Yorke JA. Chaos in a double pendulum. American Journal of Physics. 1992;60(6):491–499.

[46] D'août K, Aerts P. Kinematics and Efficiency of Steady Swimming in Adult Axolotls (*Ambystoma Mexicanum*). Journal of Experimental Biology. 1997;200(13):1863–1871. doi:10.1242/jeb.200.13.1863.

[47] Karakasiliotis K, Schilling N, Cabelguen JM, Ijspeert AJ. Where are we in understanding salamander locomotion: biological and robotic perspectives on kinematics. Biological Cybernetics. 2013;107(5):529–544. doi:10.1007/s00422-012-0540-4.
