# Supplementary material for: Balancing central control and sensory feedback produces adaptable and robust locomotor patterns in a spiking, neuromechanical model of the salamander spinal cord
Source: PLoS Comput Biol. 2025 Jan 21;21(1):e1012101. doi: 10.1371/journal.pcbi.1012101 (PMC11771899; doi:10.1371/journal.pcbi.1012101)
Supplement: Attachment — Submitted filename: Response to Reviewers.pdf [file pcbi.1012101.s014.pdf]

Authors' Response to Reviews of

Balancing central control and sensory feedback produces adaptable and robust locomotor patterns in a spiking, neuromechanical model of the salamander spinal cord

A. Pazzaglia, A. Bicanski, A. Ferrario, J. Arreguit, D. Ryczko, A. Ijspeert
PLOS Computational Biology,

RC: ***Reviewers' Comment***, AR: Authors' Response, AC: Authors' Changes,

We sincerely thank both reviewers for their time as well as for their useful comments and suggestions. In the following text, **RC** indicates the reviewers' comments, AR indicates our responses, AC describes our changes to the paper.

1. Reviewer #1

RC: ***Thank you for addressing my concerns. I have one final question. Most of your responses, like on neuronal types, have narrowed the research down specifically to salamanders. Granted a special species. The fundamental organization, or modeling concept, has not been completely validated experimentally, and has been challenged/provided with alternative solutions, like Lindén et al. 2022. It would be helpful to discuss if your conclusions still stand should the alternative locomotion control solution is true, or the alternative is simply impossible here.***

AR: We agree that several aspects of the locomotor circuits in vertebrates remain elusive. On the other hand, many properties related to their fundamental organization have been well characterized and established in the literature [1]. As discussed in previous responses to the reviewer, the core assumptions used in this model (namely the presence of balanced recurrent excitation and commissural inhibition) are shared by all known CPG architectures and models [1]. The main contribution of the study mentioned by the reviewer was the finding that population activity in correspondence with the lumbar spinal cord of the turtle covers all phases, rather than oscillating between two alternating states [2]. The recording area covered three spinal segments (D8, D9, D10), comprising the CPG oscillators for all the limb degrees of freedom (hip, knee, ankle), the corresponding axial oscillators, as well as the limb and axial sensory neurons. If we were to mimic the same recording in our model, the recording area would include the pre-girdle axial neurons, post-girdle axial neurons, and the limb oscillator neurons with various delays for the different degrees of freedom of the limbs (see section "Electro-mechanical transduction" in the main text). Consequently, despite its simplicity, our model would also almost uniformly cover all the neural phases. Furthermore, our model focuses on the core neural classes of spinal locomotor circuits. Including additional neural types (e.g.; V1, V0v, V3 interneurons and other sensory modalities) would likely further contribute to spread the phases covered by neurons during the locomotor rhythms. Ultimately, we do not identify a fundamental contradiction between our model and the one proposed by [2] and we think that some of the conclusions drawn in our study could be useful to improve the understanding of the locomotor circuits regardless of the framework chosen to describe them.

AC: We added a discussion of this point in the main text, see section "Limitations of the model"

RC: ***I otherwise recommend this article for publication.***

2. Reviewer #2

RC: ***I would like to thank the authors for their diligent efforts in addressing the reviewers' concerns. The addition of detailed analyses in response to Reviewer 1's Comment C1, particularly the inclusion of "Simulating Fictive Locomotion" in Supplementary File S5, appears to have addressed his/her concerns. The revisions, especially to the title, Introduction, and overall structure, have greatly improved the clarity of the authors' messages and have significantly strengthened the manuscript. This study makes a valuable contribution to understanding the neuronal basis of movement in vertebrates and will undoubtedly inspire future research.***

References

[1] Grillner S, El Manira A. Current Principles of Motor Control, with Special Reference to Vertebrate Locomotion. Physiological Reviews. 2020;100(1):271–320. doi:10.1152/physrev.00015.2019.

[2] Lindén H, Petersen PC, Vestergaard M, Berg RW. Movement is governed by rotational neural dynamics in spinal motor networks. Nature. 2022;610(7932):526–531. doi:10.1038/s41586-022-05293-w.
